# Prevalence of *Salmonella* Infection in Dogs in Maiduguri, Northeastern Nigeria

**DOI:** 10.1155/2014/392548

**Published:** 2014-10-23

**Authors:** Saleh Mohammed Jajere, Samson Amali Onyilokwu, Nuhu Bala Adamu, Naphtali Nayamanda Atsanda, Adamu Saleh Saidu, Shuaibu Gidado Adamu, Fatima Bukar Mustapha

**Affiliations:** ^1^Department of Veterinary Public Health and Preventive Medicine, Faculty of Veterinary Medicine, University of Maiduguri, PMB 1069, Maiduguri, Borno State, Nigeria; ^2^Veterinary Microbiology Research Laboratory, Department of Veterinary Microbiology and Parasitology, Faculty of Veterinary Medicine, University of Maiduguri, PMB 1069, Maiduguri, Borno State, Nigeria; ^3^Department of Veterinary Public Health and Preventive Medicine, Faculty of Veterinary Medicine, Ahmadu Bello University, PMB 1096, Zaria, Kaduna State, Nigeria

## Abstract

The prevalence and antimicrobial sensitivity of *Salmonella* from dogs in Maiduguri Metropolis were determined using standard bacteriological methods to assess the risk of possible transmission of *Salmonella* infection from dogs to humans. Of 119 samples, *Salmonella* was isolated from 52 (43.7%). Males had higher prevalence of 50.0% compared with 34.7% in females (*P* < 0.05). Dogs older than 24 months had higher prevalence of 61.0% and the lowest was seen in dogs aged 13–24 months (*P* < 0.05). The prevalence of 31.8%, 41.2%, and 58.8% was observed in dogs aged 3–6, 10–12, and 7–9 months, respectively. High prevalence of 49.5% was observed in Mongrels, while Terrier and Alsatian breeds had 30.0% and 8.3%, respectively. *Salmonella* isolates from Alsatian and Terrier breeds showed about 100% susceptibility to all the tested antimicrobials. Higher percentage of the *Salmonella* isolates from Mongrels also showed susceptibility to ciprofloxacin (89.7%), amoxicillin (87.6%), vancomycin (86.6%), and chloramphenicol (84.5%). However about 50% of these isolates showed resistance to ofloxacin. The carrier status of *Salmonella* is high among dogs especially Mongrels. Therefore good environmental hygiene, discouraging straying coupled with feeding of dogs with properly cooked and uncontaminated feeds was recommended to mitigate risk of human salmonellosis.

## 1. Introduction

Salmonellosis is a broad term applied to enteric infections caused by a group of gram-negative bacteria of the genus* Salmonella*, belonging to the family Enterobacteriaceae [1].* Salmonella enterica*, the causative agent of salmonellosis, is known to be a primary inhabitant of the gastrointestinal tract of numerous animal species including humans and mostly affects a wide host range with the exception of a few* Salmonella *serovars that are host-specific:* Salmonella *Typhi in humans,* S. *Gallinarum and* S.* Pullorum in poultry, and* S.* Dublin in cattle [1]. They are common contaminants of wide range of food, eggs, vegetables, and water and are therefore considered as the most common causes of foodborne zoonotic infection worldwide [2]. Consequently, they are recognized as a major public health problem in both developed and developing countries [3]. Feaces of nearly all-animal species including dogs may serve as a potential source of* Salmonella* infection to humans and even to other animals [1]. Several studies have documented the isolation of* Salmonella* spp. from healthy dogs and subsequent transmission of the infection to humans [4–7]. Other studies have recognized shedding of* Salmonella* by dogs as a possible source of infection for dog owners and their communities [8, 9]. Dogs generally remain resistant to* Salmonella* infections and infected ones may remain carriers and feacal shedding therefore may serve as sources of infection to man and other animals [8]. Due to the increase in dog keeping among the elites especially those living within the metropolitan cities in Nigeria, there is an increased risk of transmission of* Salmonella* infection to humans. There is paucity of information regarding the role of dogs as sources of* Salmonella *infection to humans in Maiduguri Metropolis and therefore this study aims to determine the carrier status of* Salmonella *in dogs and their role as potential sources of infection to humans.

## 2. Materials and Methods

### 2.1. Study Area

Maiduguri is located in the Sahel Savannah region of Northeastern Nigeria at 11°50^I^–11.83° North Latitude and 13°09^I^–13.15° East Longitude. It shares an extensive border with Niger to the North, Chad to the Northeast, and Cameroon to the East. Its altitude is about 350 metres above sea level and has an area of 75,540.9 km^2^. Its population is about 4,171,104 with a population density of 60 people per km^2^ (2006 census figures). The climate is favourable, with a mean annual rainfall and temperature of about 650 mm and 32°C, respectively. The months of March and April are the hottest periods of the year with temperatures ranging between 30°C and 40°C. It is usually cold and dry during the harmattan, November to January being the coldest months (http://en.wikipedia.org/wiki/Maiduguri).

### 2.2. Sources, Baseline Characteristics, and Inclusion Criteria of Dogs

The dogs investigated in this study were from Maiduguri Metropolis and its environs. Samples were obtained only from dogs kept at home for security/guard or other purposes and from dogs at the Veterinary Teaching Hospital (*n* = 20), University of Maiduguri staff quarters (*n* = 11), Mairi village (*n* = 23), Gwange ward (*n* = 15), Bulunkutu Polo (*n* = 18), Custom area (*n* = 10), Gommari (*n* = 13), and Ummalari (*n* = 9) wards. A total of 119 feacal samples, comprising 70 males and 49 females, were collected from three breeds of dogs (Nigerian local breed Mongrel, Alsatian, and Terrier breed). The age of the dogs was determined by dental eruption at the time of sample collection. Only one dog was sampled per household visited. Most of the household visited had 3 to 4 dogs. A total of 97 Nigerian local breed Mongrel and 12 Alsatian and 10 Terrier breeds were sampled. All the ages of the sampled dogs ranged from <3 months to older than 2 years. All the dogs were apparently healthy dogs with only 5 (4.2%) having diarrhoea as at the time of sample collection.

### 2.3. Sample Collection and Bacteriological Analysis

Rectal swabs were collected aseptically using sterile swab sticks (Oxoid) and placed in tubes containing Carry-Blair transport medium (Oxoid, Basingstoke, UK). The samples were immediately transported ice cooled within 8 hours of collection to the Microbiology Laboratory, Faculty of Veterinary Medicine, University of Maiduguri, for further processing and analysis. All the samples collected were then inoculated into 2 mL of Selenite-Feaces-broth (Oxoid, Biotec, Suffolk, UK) for enrichment and incubated aerobically at 37°C for 24 hours. Subcultures were then made from each broth culture by streaking onto Brilliant Green Agar (BGA) (Oxoid), McConkey agar (Oxoid),* Salmonella*-*Shigella *Agar (SSA) (Oxoid), and Xylose Lysine deoxycholate (XLD) agar (Oxoid). The cultured plates were incubated at 37°C for 24–48 hours [10]. The cultured plates were then examined for the presence of typical colonies of* Salmonella *based on cultural and morphological characteristics: presumptive* Salmonella* colonies appearing colourless and nonlactose fermenting on McConkey agar, dome-shaped colonies with central black spot on XLD agar, transparent colonies with black centre on SSA, and pink colonies surrounded by a red medium were selected as presumptive* Salmonella* colonies and subjected to further biochemical tests and gram staining as described by Barrow and Feltham [11].

### 2.4. Antibiotic Sensitivity Testing

Antibiotic susceptibility pattern of isolates to various routine antibiotics was carried out using the Kirby-Bauer disk diffusion method [12] on Mueller-Hinton Agar (Oxoid) as recommended by Anonymous [13]. The susceptibility of the isolates to the following antimicrobials was determined: tetracycline (30 *μ*g), gentamycin (30 *μ*g), chloramphenicol (30 *μ*g), ciprofloxacin (10 *μ*g), ofloxacin (5 *μ*g), streptomycin (10 *μ*g), ampicillin (30 *μ*g), amoxicillin-clavulanic acid (AMC) (5 *μ*g), vancomycin (30 *μ*g), neomycin (25 *μ*g), trimethoprim (25 *μ*g), and erythromycin (15 *μ*g).

### 2.5. Statistical Analysis

Prevalence of* Salmonella *infection was expressed in percentages and proportions in Microsoft Excel version 2010 before finally importing the data into Statistical Package for Social Sciences (SPSS) software version 16.0 to determine the statistical association between dependent and independent variables. The association between infection and other risk factors was determined using Chi-square test at 5% significance level [14].

## 3. Results

Of the 119 sampled dogs, 70 (58.8%) and 49 (41.2%) were males and females, respectively; 97 (81.5%), 12 (10.1%), and 10 (8.4%) were Nigerian local breed Mongrel, Alsatian, and Terrier, respectively; 2 (1.2%), 22 (18.5%), 17 (14.3%), 17 (14.3%), 20 (16.8%), and 40 (34.5%) were from dogs aged < 3, 3–6, 7–9, 10–12, 13–24, and older than 24 months, respectively (Table 1).

Of the 119 dogs examined, 52 (43.7%) were positive for* Salmonella*. Of these (52), 50.0% and 34.7% were the male and female infection rates, respectively (Table 2). There was significant difference (*P* < 0.05) in the prevalence of* Salmonella *infection in either sex. Highest prevalence was observed in dogs aged older than 24 months with 61.0% (25/41) and except for dogs < 3 months, the lowest was seen in dogs 13–24 months with 15.0% (3/20). While the prevalence of 31.8%, 41.2%, and 58.8% was observed in dogs aged 3–6, 10–12, and 7–9 months in this order of infection rates (Table 2). There was statistically significant difference (*P* < 0.05) in the prevalence of* Salmonella* infection across the age groups. Highest prevalence of 49.5% (48/97) was observed in the Nigerian local breed Mongrel, while Terrier and Alsatian breeds had 30.0% (3/10) and 8.3% (1/12), respectively (Table 2). This was statistically significant (*P* < 0.05).

The antimicrobial sensitivity testing of the* Salmonella* isolates reveals that* Salmonella* isolates from the Terrier and Alsatian breeds had high susceptibility to all the antimicrobials used, with small pockets of resistance (Table 3). While a higher percentage of the* Salmonella *isolates from the Mongrels showed susceptibility to ciprofloxacin (89.7%), amoxicillin (87.6%), vancomycin (86.6%), chloramphenicol (84.5%), trimethoprim (81.4%), ampicillin (80.4%), neomycin (77.3%), and gentamycin (71.1%) (Table 3), however, about 50% of the isolates from Mongrels were resistant to ofloxacin.

## 4. Discussion

Dogs as carriers of* Salmonella *spp. worldwide have been incriminated as the potential sources of* Salmonella *infection to humans especially children due to close contact and interaction that exists between them [15]. Cases of* Salmonella *transmission from dogs to humans resulting into severe infection in humans have been documented [16]. It was also reported that the intestinal carriage of* Salmonella* by dogs is more common than the prevalence of clinical disease [9]. The frequency of feacal isolation of* Salmonella* spp. from clinically healthy dogs was reported to be between 0.0% and 43.0% [9, 15], while other studies reported prevalence of* Salmonella* in feacal samples from clinically healthy or hospitalized dogs to range from 1% to 36% [17]. However, recent studies suggested that the prevalence is probably decreasing because more pets are fed commercially processed foods [17]. In the present study,* Salmonella* was isolated from 52 (43.7%) of the dogs examined. This prevalence is within the reported 0.0% and 43.0% range of prevalence of* Salmonella* isolated from clinically healthy dogs [9, 15]. However, this finding is consequently higher than the results obtained from other parts of Nigeria and other countries in the world. In similar studies by Britt et al. [18] in Vom, Nigeria reported that 18.3% of 120 domestic dogs were excreting* Salmonella* serotypes. Another study by Khan [19] in Sudan reveals that about 23.5% of 442 dogs were positive for* Salmonella*. The differences in the sample sizes, period of study, type of feacal samples, geographical areas, and isolation methods employed in the various studies above may account for the differences in the prevalence and may all affect the prevalence [20]. The high prevalence of 43.7% in dogs in Maiduguri Metropolis might be associated with poor management conditions, compromised good and hygienic environmental standards, contaminated kennels, and food sources. Mongrels in the study area are mostly kept under less restraint and are left to wander and scavenge for food. This makes them highly exposed to* Salmonella *contaminated food materials in the environment (Table 2).

The absence of* Salmonella* in dogs within the age of <3 months could be attributed to the small number of dogs from which samples were obtained (Table 2). It could also result probably from the protection by the maternal antibodies in dogs within this age bracket. There is seemingly high prevalence of* Salmonella* in dogs within the age groups of 3–6, 7–9, and 10–12 months. This is in concordance with the works of Britt et al. [18] in Vom, Plateau State, that* Salmonella* infections are acquired during the early stages of life in dogs. This could result from the low resistance of the immune system to infection by* Salmonella* during this stage of life in dogs. The low prevalence of 15.0% observed in dogs within 13–24 months could be the result of risen immunity and possibly good environmental and management conditions provided by the pet owners. Morse et al. [4] reported that feacal shedding could last for a period of 6 weeks and this could possibly be the reason why the prevalence was lower in dogs within this age bracket. A high prevalence of 61.0% observed in dogs within the age of >24 months might possibly be attributed to the fact that older dogs are usually immunosuppressed and therefore at high risk of infection [17]. This renders dogs asymptomatic carriers of the infection and consequently contaminates human environment, food, and water resulting in outbreaks of human salmonellosis.

Males had higher prevalence of* Salmonella* infection compared with females (50.0% versus 34.7%). This could be due to the differing number of samples in each sex (Table 2). According to breeds, Nigerian local breed Mongrel showed the highest prevalence of 49.5%. This is in agreement with the work of Britt et al. [18] who highlighted the fact that due to the poor management and compromised sanitary conditions, a large number of Mongrels tend to acquire the infection and shed the organism in the environment. A large population of the Mongrels in the study area is normally kept under less restraint and left to wander around wider range of distances scavenging for food and therefore get exposed to many contaminated materials compared with the Terrier and Alsatian breeds kept under good hygienic conditions. Scavengers are likely to harbor more* Salmonella *serovars than nonscavengers kept under strict restraint and good hygienic conditions [1]. Compromised sanitary conditions could lead to feacal contamination of the food and water of the Mongrels in the environment and therefore could serve as a source of infection in this breed. Finley et al. [21] also observed that when dogs are fed with Salmonella-contaminated feed, raw food, and commercially prepared dry foods, they can become infected and consequently shed the organism in their feaces to contaminate the environment, domestic animals, other dogs, and even man. The local breed Mongrels are provided with poor environmental conditions and are less cared for compared with the exotic breeds of Terrier and Alsatian. The Alsatian and Terrier breeds are kept at home mostly by the elites (restrained) and fed with commercial food and this makes them less exposed to* Salmonella* infection compared with the Mongrels (Table 2).

The antimicrobial sensitivity testing results reveal that high percentages of the* Salmonella* isolates from the Alsatian and Terrier breeds were susceptible to the antimicrobials used, while a high percentage of the isolates from Mongrels showed susceptibility to ciprofloxacin (89.7%), amoxicillin-clavulanic acid (AMC) (87.6%), vancomycin (86.6%), and chloramphenicol (84.5%). However, about 50% of these isolates were resistant to ofloxacin (Table 3). In the past decades, the emergence of antibiotic resistant strains of* Salmonella* has become a major public health concern. Mongrels are more likely to harbor* Salmonella *serovars due to their scavenging nature and therefore are more likely to be treated with antibiotics than the exotic breeds in the study area. Alsatian and Terrier breeds are mostly restricted and fed at home and are not allowed to scavenge or stray in the environment. This makes them less likely to acquire* Salmonella *infection and therefore not likely to be treated with antibiotics. Indiscriminate use of antibiotics in animals could be responsible for the emergence of antimicrobial resistant strains of bacteria [1]. Therefore, the use of antibiotics should be well regulated and used only when it is indicated. Ciprofloxacin and amoxicillin-clavulanic acid (AMC) could be useful antibiotics in the treatment of majority of Salmonellosis cases as revealed by their effectiveness against a high percentage of* Salmonella *isolates from the study area.

In conclusion, this study revealed a high* Salmonella* carrier status in dogs kept in households in Maiduguri Metropolis, Nigeria. Mongrels had high Salmonella carrier status compared with the other breeds in the study area. Most of the households visited keep Mongrels, where they are left to wander and scavenge for food in the environment and only return back home in the evening. This has public health significance as dogs may pose a risk to humans where close contact between dogs and their owners occurs in households and children and immunosuppressed individuals may be particularly at risk [6]. As the infection is acquired during the early stage of their life, pet keepers or dog keepers should maintain a high personal and environmental standard hygiene of the dogs to mitigate the potential zoonotic transmission to humans. Properly cooked and uncontaminated feed should be fed to dogs and scavenging and straying of dogs should be discouraged. It is recommended that complete bacteriological identification be carried out on the isolates to determine the serotypes found in the study area.

## Figures and Tables

**Table 1:** Baseline characteristics of the sampled dogs in Maiduguri, Nigeria (*n* = 119).

Risk factors	Number sampled	% of total population
Sex		
Male	70	58.8
Female	49	41.2
Age (months)		
<3	2	1.7
3–6	22	18.5
7–9	17	14.3
10–12	17	14.3
13–24	20	16.8
>24	41	34.5
Breeds		
Mongrel	97	81.5
Alsatian	12	10.1
Terrier	10	8.4
Overall total	**119**	**100**

**Table 2:** Prevalence of *Salmonella* among exotic and local breeds of dogs according to sex, age, and breeds in Maiduguri, Nigeria (*n* = 119).

Risk factors	Number sampled	Number (%) positive
Sex		
Male	70	35 (50.0)^a^
Female	49	17 (34.7)
Age (months)		
<3	2	—
3–6	22	7 (31.8)^a^
7–9	17	10 (58.8)
10–12	17	7 (41.2)
13–24	20	3 (15.0)
>24	41	25 (61.0)
Breeds		
Mongrel	97	48 (49.5)^a^
Alsatian	12	1 (8.3)
Terrier	10	3 (30.0)
Total	**119**	**52 (43.7)**

^a^Statistically significant difference in infection rates (*P* < 0.05).

**Table 3:** Antibiotic susceptibility pattern of *Salmonella* isolates from exotic and local breeds of dogs in Maiduguri, Nigeria (*n* = 119).

Antibiotic (amount in *μ*g)	Mongrel^a^	Alsatian^b^	Terrier^c^	Total
	Number (%) *Resistant *	Number (%) *Resistant *	Number (%) *Resistant *	Number (%) *Resistant *
Gentamycin (30)	28 (28.9)	0 (0.0)	0 (0.0)	28 (23.5)
Amoxicillin-clavulanic acid (5)	11 (11.3)	0 (0.0)	0 (0.0)	11 (9.2)
Erythromycin (15)	32 (33.0)	1 (8.3)	0 (0.0)	33 (27.7)
Ciprofloxacin (10)	10 (10.3)	0 (0.0)	0 (0.0)	10 (8.4)
Ampicillin (30)	19 (19.6)	2 (16.0)	0 (0.0)	21 (17.6)
Streptomycin (10)	30 (30.9)	0 (0.0)	0 (0.0)	30 (25.2)
Tetracycline (30)	36 (37.1)	0 (0.0)	0 (0.0)	36 (30.3)
Chloramphenicol (30)	15 (15.6)	0 (0.0)	0 (0.0)	15 (12.6)
Ofloxacin (5)	48 (49.5)	1 (8.3)	0 (0.0)	49 (41.2)
Vancomycin (30)	13 (13.4)	0 (0.0)	0 (0.0)	13 (10.9)
Neomycin (25)	22 (22.7)	0 (0.0)	0 (0.0)	22 (18.5)
Trimethoprim (25)	18 (18.6)	0 (0.0)	0 (0.0)	18 (15.1)

^a^Mongrels (*n* = 97); ^b^Alsatian (*n* = 12); ^c^Terrier (*n* = 10).
